# Random Walk on T-Fractal with Stochastic Resetting

**DOI:** 10.3390/e26121034

**Published:** 2024-11-29

**Authors:** Xiaohan Sun, Anlin Li, Shaoxiang Zhu, Feng Zhu

**Affiliations:** 1 School of Mathematical Science, Jiangsu University, Zhenjiang 212013, China; njsunxiaohan@126.com (X.S.); 2540624784@qq.com (A.L.); 2 School of Mechanical Engineering, Jiangsu University, Zhenjiang 212013, China; zhushaoxiang6@gmail.com

**Keywords:** random walk, T-fractal, stochastic resetting, generating function, first passage time

## Abstract

In this study, we explore the impact of stochastic resetting on the dynamics of random walks on a T-fractal network. By employing the generating function technique, we establish a recursive relation between the generating function of the first passage time (FPT) and derive the relationship between the mean first passage time (MFPT) with resetting and the generating function of the FPT without resetting. Our analysis covers various scenarios for a random walker reaching a target site from the starting position; for each case, we determine the optimal resetting probability $\gamma^{*}$ that minimizes the MFPT. We compare the results with the MFPT without resetting and find that the inclusion of resetting significantly enhances the search efficiency, particularly as the size of the network increases. Our findings highlight the potential of stochastic resetting as an effective strategy for the optimization of search processes in complex networks, offering valuable insights for applications in various fields in which efficient search strategies are crucial.

## 1. Introduction

In recent years, research on random walks with stochastic resetting has garnered significant interest due to its applications in diverse fields, including search processes [1,2,3,4], network theory [5,6,7,8], and optimization [9,10]. Stochastic resetting is a process in which a random walker is periodically reset to a specific node or state; it modifies the inherent dynamics of random walks, offering advantages in terms of the mean first passage times and exploration efficiency. This concept was first comprehensively studied in the context of complex networks in 2020, where the researchers analyzed random walks on networks with a single resetting node, laying foundational models that have informed subsequent studies [11].

Following this initial study, stochastic resetting on complex networks was extended to more complex settings. Such research has explored multiple resetting nodes, which provide a more flexible framework by allowing resets to occur at various points across the network. Different theoretical approaches to the modeling of multiple resetting nodes have been proposed, broadening our understanding of reset mechanisms and their effects on the network dynamics [12,13]. Furthermore, studies have expanded the resetting process to include node-dependent and time-dependent resetting protocols. These approaches allow the reset probabilities to vary by node or over time, respectively, offering refined control over the walker’s behavior and enabling the adaptation of the resetting process to network-specific attributes [14,15]. Another notable development is the introduction of first passage resetting, where reset events are triggered when the walker reaches any designated observable node, presenting a new perspective on dynamic resetting rules [16].

Despite these advancements, there remains an opportunity to explore novel reset mechanisms and adapt stochastic resetting to specialized network structures, such as fractal-like topologies, which are known for their unique scaling properties and hierarchical structures. Among these, the T-fractal—a self-similar, scale-invariant structure—has attracted attention for its analytical tractability and applications in fields such as wireless networks [17,18,19], biology [20,21], and material science [22,23,24]. However, while these fractal networks have practical value in modeling hierarchical or modular systems, background information regarding their specific applications and value is limited in the current literature [25].

In this study, we examine the intricate behavior of random walks on a specific class of fractals known as T -fractals, which belong to the broader family of tree-like fractals. Tree-like fractals are constructed through an iterative process, governed by a positive integer parameter (m), and they exhibit self-similar patterns at different scales [26]. The T-fractal, in particular, emerges as a special case when (m = 1), exhibiting unique structural properties that make it an ideal candidate for the study of random walks and their responses to stochastic resetting.

The investigation of random walks on fractals has long been a topic of interest due to their ability to model diffusion processes in complex geometries and heterogeneous media [27,28]. The T-fractal’s hierarchical structure, characterized by its branching nature and increasing complexity with each generation, provides a rich backdrop for the exploration of the ways in which the fractal dimension and other geometrical features affect the random walk dynamics.

Our primary focus is on deriving analytical expressions for the mean first passage time (MFPT) of random walks on the T-fractal, with and without stochastic resetting [29]. Specifically, we consider various scenarios, including walks starting from the outermost nodes, the central node, and randomly selected nodes based on the stationary distribution [30]. For each scenario, we employ generating functions as a powerful tool to address the recursive nature of the problem and obtain closed-form solutions for the MFPT [31].

Furthermore, we extend our analysis to include the effect of stochastic resetting on these random walks. By incorporating resetting events that occur at a fixed rate, we investigate how this mechanism modifies the MFPT and determine the optimal resetting probability that minimizes the MFPT. Such findings offer valuable insights into the design of efficient search strategies and the dynamics of diffusion-limited processes in fractal-like environments [32].

In summary, this work contributes to the understanding of random walks on complex networks by providing a comprehensive analysis of random walks and stochastic resetting on the T-fractal. Our results not only deepen the theoretical foundation of this field but also have potential implications for various applications, including target searches in complex media, diffusion-controlled reactions, and optimization problems on fractals.

## 2. Network Model and Some Properties

### 2.1. The Construction of Tree-like Fractals

Firstly, let us examine the overarching network architecture of the T-fractal, which is categorically known as a tree-like fractal [33]. These fractals are constructed through an iterative process and possess a fundamental positive integer parameter, *m*, which plays a pivotal role in their formation. Let $F_{t}$ signify the tree-like fractal at a particular generation *t* (where *t* is a non-negative integer).

The methodology employed in their creation commences with a rudimentary starting point involving two nodes that are linked by a solitary edge, which is denoted as $F_{0}$. This foundational configuration serves as the building block for the more complex iterations that follow. For *t* values that are greater than or equal to 1, the fractal $F_{t}$ is pieced together by inserting a new vertex at the midpoint of each existing edge within $F_{t-1}$. This innovative vertex is then seamlessly connected to both terminal points of the original initial edge. This process continues as m additional edges are appended to the nodes that were most recently introduced into the structure.

**Figure 1:**
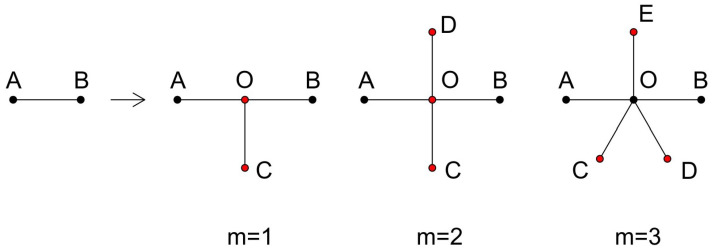
Iterative construction of tree-like fractals from generation 0 to generation 1. The next generation is created by adding one vertex O between nodes A and B; we then add m edges to O.

**Figure 2:**
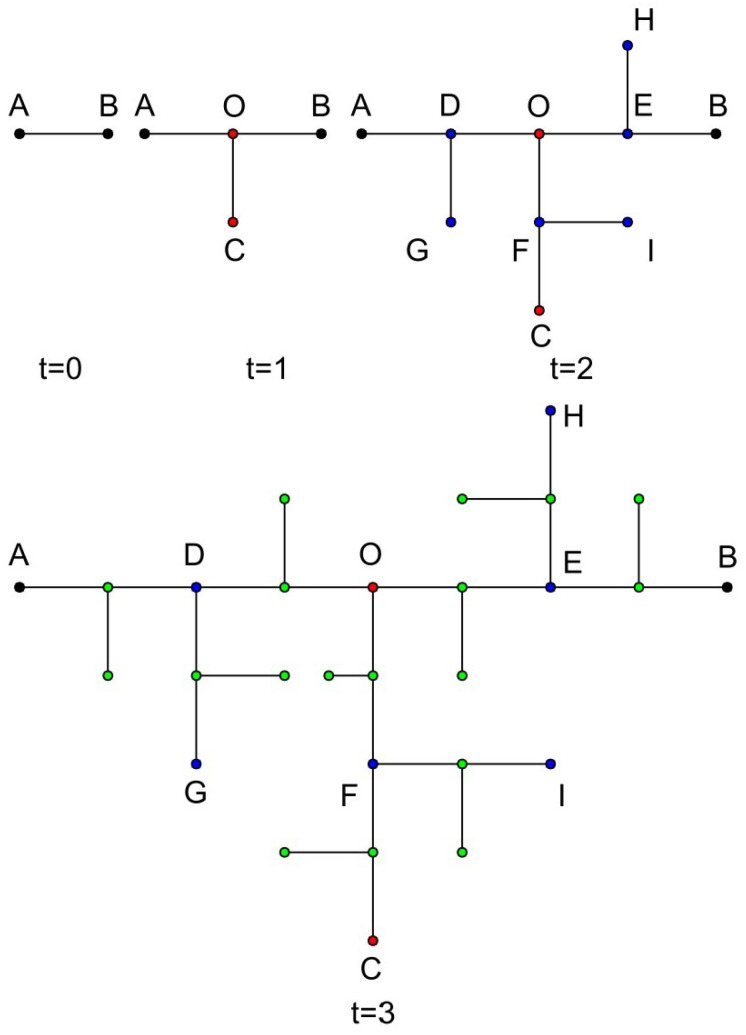
The growth process of the T-fractal (a special case of tree-like fractals when m = 1). The nodes in black were generated in generation 0, red nodes in generation 1, blue nodes in generation 2, green nodes in generation 3, and so on.

For visual clarity, Figure 1 provides a graphical representation of the initial generation network’s structure when various values of m are chosen. Figure 2 further elucidates the composition of each generation within the tree-like fractal when m is set to 1, a scenario also referred to as the T-fractal. In this figure, the nodes that are generated at disparate generations are depicted using an array of colors, which serves as a helpful tool in comprehending the composite nature of the T-fractal. In this visual depiction, the black color is utilized to designate nodes that are born in the zero generation, while red is employed to mark the nodes from the first generation. The blue color is assigned to represent the nodes from the second generation, followed by green for the third generation, and so on, thereby providing a clear visual illustration of the fractal’s generational growth.

**Figure 3:**
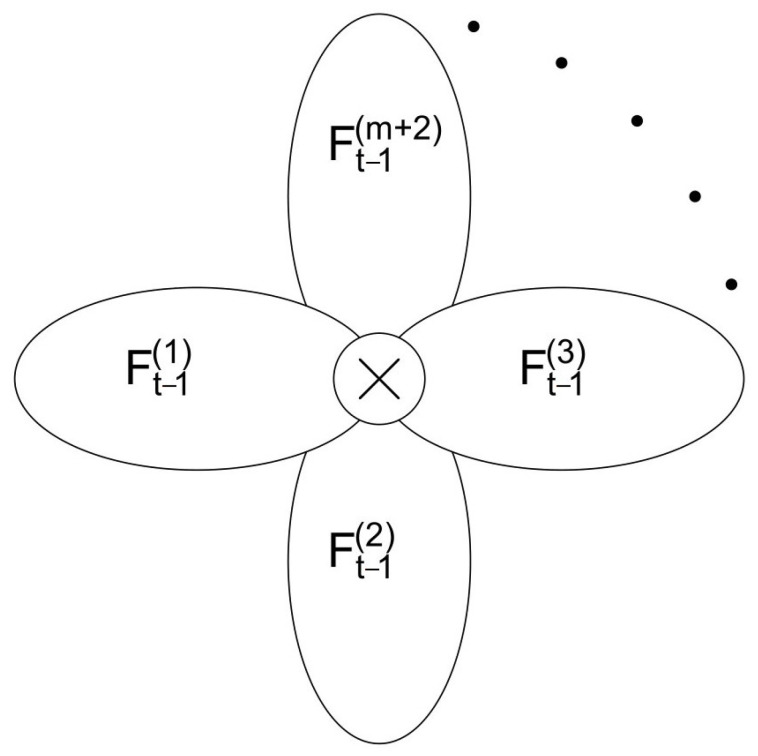
Another approach to obtaining a tree-like fractal. $F_{t}$ can be regarded as m+2 copies of $F_{t-1}$, connecting the outermost vertex and the innermost vertex.

It is worth mentioning that there is another method to construct tree-like fractals. From the perspective of the first generation, the central node O of the first generation is regarded as the innermost node, and the node farthest from the center is regarded as the outermost node. Thus, it can be regarded as m + 2 copies, connecting the outermost vertex and the innermost vertex, as shown in Figure 3.

### 2.2. Some Basic Properties of Tree-like Fractals

According to the tree-like fractal construction method, the number of edges in each generation of the network is m+2 times that of the previous generation. Therefore, we can easily conclude that the number of edges in the t-th generation of the tree-like fractal is $E_{t}={\left(m+2\right)}^{t}$. Given that the number of vertices in the tree structure is one greater than the number of edges, we can conclude that the number of vertices in the t-th generation of the network is $N_{t}={\left(m+2\right)}^{t}+1$.

In addition, after each iteration, the diameter of the fractal (i.e., the distance from the innermost node to the outermost node) doubles. As the number of network edges increases by m+2 times, we can conclude that the tree dimension is $d_{f}=\frac{\ln(m+2)}{\ln2}$. In addition, it has been found in [34] that, in the spectral for any two nodes *i* and *j* in the current generation, the mean first passage time (MFPT) from node *i* to node *j* will increase by 2(m+2) times in the next generation. Therefore, the dimension of the tree walk is

$$d_{r}=\frac{\ln[2(m+2)]}{\ln2}=1+d_{f},$$

and the spectral dimension is

$$d=\frac{2d_{f}}{d_{r}}=2\frac{\ln(m+2)}{\ln[2(m+2)]}=\frac{2d_{f}}{1+d_{f}}.$$

## 3. Random Walk on T-Fractal

In this section, we analyze the random walk on the T-fractal, whose structure is shown in Figure 2. The T-fractal is a specific case of a tree-like fractal when m=2. The analytical framework presented here can be extended to other values of *m* as well. We first investigate the mean first passage time (MFPT) for random walks between the two outermost points (specifically, from point A to point B). Following this, we study the MFPT between two other boundary points (such as from point D to point E); finally, we derive the MFPT for a random walk starting from the central point O. In this analysis, we employ the generating function method, which is particularly effective in addressing the complexities of random walks on fractal structures.

It is important to acknowledge that several previous studies have also examined random walks on the T-fractal, with results that are closely aligned with those given in Section 3 of this work. Key contributions include [35,36,37,38]. Unlike these works, our approach leverages the generating function method to provide a more comprehensive and generalized analysis of the random walk problem on the T-fractal.

### 3.1. Random Walk from A to B

In this subsection, we study the properties of the first passage time (FPT) from A to B (see Figure 2). In order to calculate the generating function of the FPT, we set A as the starting point and B as the absorption domain, assuming that the FPT from A to B is a random variable $T_{A\to B}\left(t\right)$. We let P(t,n) denote the first passage probability (FPP), which is the probability that a random walker, starting from an initial node *i*, reaches a target node *n* for the first time exactly at time *t*.

The FPP P(t,n) can be defined as

(1) $$P\left(t,n\right)=\mathrm{Pr}\left(T_{n}=t\right),$$

where $T_{n}$ is the first passage time to the target node *n*. Suppose that the generating function of $T_{A\to B}\left(t\right)$ is $\Phi_{T}^{A\to B}\left(t,z\right)$. Therefore, according to the definition of the generating function (see Appendix A), we can obtain the probability generating function of $T_{A\to B}\left(t\right)$ as

(2) $$\Phi_{T}^{A\to B}\left(t,z\right)=\sum_{n=0}^{+\infty }P\left(t,n\right)z^{n},$$

where t represents the generation of the T-fractal.

Now, we denote by Ω the set of nodes in generation 1, which includes nodes A,B,C,O. For any path π starting at node A and reaching B, we use $v_{i}$ to denote the node in $F_{t}$ that reaches B at time *i*. In this way, we can denote the process as $\pi =(v_{0}=A,v_{1},\cdots ,v_{T_{A\to B}}\left(t\right)=B)$. Moreover, we introduce the observable $\tau_{i}=\tau_{i}\left(\pi \right)$ to represent the time taken to reach B for the i-th time for any node in Ω along the path π. The time can be defined in the following way:

$$\tau_{0}\left(\pi \right)=0,$$

(3) $$\tau_{i}\left(\pi \right)=argmin\left\{k:k>\tau_{i-1},v_{\tau_{i}}\in \Omega ,v_{\tau_{i}}\neq v_{\tau_{i-1}}\right\}.$$

Moreover, we call $N=argmin\{i:v_{\tau_{i}}=B\}$. In fact, *N* stands for the first passage time in the random walk in generation 1 (or set Ω). Thus, if we only consider the random walk in the set Ω, the path π can be simplified as

(4) $$\sigma \left(\pi \right)=\left(v_{\tau_{0}},v_{\tau_{1}},\cdots ,v_{\tau_{N}}\right),$$

The simplified path σ(π) only includes nodes that were generated in generation 1, and the time interval between the two steps in σ(π) is stochastic. Thus, we can denote the random variable $\eta_{i}$ via the time interval between $v_{\tau_{i-1}}$ and $v_{\tau_{i}}$, i.e.,

(5) $$\eta_{i}=\tau_{i}-\tau_{i-1}.$$

According to the second T-fractal construction method, $F_{t}$ can be regarded as being composed of three $F_{t-1}$, and there are two points among A, B, C, and O that are the endpoints of $F_{t-1}$. Therefore, we can infer that $\eta_{i}$ has the same distribution, they have the same distribution as $T_{A\to B}\left(t-1\right)$, and the generating function is $\Phi_{T}^{A\to B}\left(t-1,z\right)$. Moreover, *N* is the FPT in the first generation, and its generating function is $\Phi_{T}^{A\to B}\left(1,z\right)$. According to the properties of the generating function (see Appendix A) and Equation (6), we can deduce that the generating function of $T_{A\to B}\left(t\right)$ satisfies

(6) $$T_{A\to B}\left(t\right)=\tau_{N}-\tau_{0}=\sum_{i=1}^{N}\eta_{i}.$$

(7) $$\Phi_{T}^{A\to B}\left(t,z\right)=\Phi_{T}^{A\to B}\left(1,\Phi_{T}^{A\to B}\left(t-1,z\right)\right).$$

As for the initial condition $\Phi_{T}^{A\to B}\left(1,z\right)$, it can be derived using a transition probability matrix for random walks on the T-fractal in generation 1; see Appendix B. The result is

(8) $$\Phi_{T}^{A\to B}\left(1,z\right)=\frac{z^{2}}{3-2z^{2}}.$$

By solving Equation (7) with the initial condition in Equation (8), we obtain

(9) $$\Phi_{T}^{A\to B}\left(t,z\right)=\frac{z^{2^{t}}}{{\left(\frac{27-36z^{2}}{3-2z^{2}}\right)}^{2^{t-1}}}\times \frac{{\left(3-2z^{2}\right)}^{2}}{27-36z^{2}}.$$

Now, we calculate the mean first passage time (MFPT) of the random walk starting from node A and ending at node B. We denote it as $E(T_{A\to B}\left(t\right))$, which is the mathematical expression for the random variable $T_{A\to B}\left(t\right)$. We can derive it by taking the derivatives on both sides of Equation (7) and using z=1 (see Appendix C). We obtain

(10) $$E\left[T_{A\to B}\left(t\right)\right]=6^{t}.$$

### 3.2. Random Walk from D to E

We now use a similar method to calculate the mean first passage time from nodes D to E. We suppose that the random variable $T_{D\to E}\left(t\right)$ denotes the first passage time from nodes D to E in generation *t* (t≥2), where the starting point is D and the end domain is E. Let $\Omega_{2}$ denote the node in generation 2. As with the method used to calculate the MFPT from A to B, we can only consider the random walk in generation 2, and we use $v_{i}$ to denote the time interval between the random walks in $\Omega_{2}$. Thus, the expression $T_{D\to E}\left(t\right)$ can also be written as

(11) $$T_{D\to E}\left(t\right)=\sum_{i=1}^{N}v_{i}.$$

We suppose that the generating function of the random variable $T_{D\to E}\left(t\right)$ is $\Phi_{T}^{D\to E}\left(t,z\right)$. Thus, in this situation, the generating function of random variable N is $\Phi_{T}^{D\to E}\left(2,z\right)$, since N stands for the random walk from nodes D to E in generation 2. Now, we analyze the generating function of the random variable $v_{i}$. It is obvious that $v_{i}\left(i=1,2,\cdots \right)$ has an identical distribution. For each random walk in generation *t*, the time interval between the nodes in generation 2 can be seen as a random walk from A to B in generation t−2. Regardless of the two nodes in generation 2, there are only two nodes at the edge of the random walk. Therefore, we can conclude that the generating functions of $v_{i}\left(i=1,2,\cdots \right)$ are $\Phi_{T}^{A\to B}\left(t-2,z\right)$. Thus, using Equation (11) and the properties of the generating function (see Appendix A, Equation (A7)), we can conclude that

(12) $$\Phi_{T}^{D\to E}\left(t,z\right)=\Phi_{T}^{D\to E}\left(2,\Phi_{T}^{A\to B}\left(t-2,z\right)\right).$$

The initial condition $\Phi_{T}^{D\to E}\left(2,z\right)$ can also be calculated by using the generating function of the matrix; see Appendix B. The result is

(13) $$\Phi_{T}^{D\to E}\left(2,z\right)=\frac{z^{2}}{6-5z^{2}}.$$

Similarly to the above method, we apply derivation on both sides of Equation (12); see Appendix C. We thus obtain the MFPT of the random walk from D to E as follows:

(14) $$E\left[T_{D\to E}\left(t\right)\right]=2\cdot 6^{t-1}.$$

### 3.3. Random Walk from O to B

Similarly to the analysis of the above two situations, we can also use the generating function to calculate the MFPT from nodes O to B, where O, as the innermost node, is the starting point of the random walk, and B is the end domain. We use the random variable $T_{O\to B}\left(t\right)$ to denote the FPT from O to B in generation *t*. The generating function of $T_{O\to B}\left(t\right)$ is denoted as $\Phi_{T}^{O\to B}\left(t,z\right)$. Similarly to the above method, we have

(15) $$T_{O\to B}\left(t\right)=\sum_{i=1}^{N}\eta_{i}.$$

where $\eta_{i}$ is the time interval between the random walks in generation 1. The generating function of random variable N is $\Phi_{T}^{O\to B}\left(1,z\right)$, and the generating function of $\eta_{i}$ is $\Phi_{T}^{A\to B}\left(t-1,z\right)$. Thus, we can obtain the following recursion:

(16) $$\Phi_{T}^{O\to B}\left(t,z\right)=\Phi_{T}^{O\to B}\left(1,\Phi_{T}^{A\to B}\left(t-1,z\right)\right).$$

The initial condition is (see Appendix B)

(17) $$\Phi_{T}^{O\to B}\left(1,z\right)=\frac{z}{3-2z^{2}}.$$

By applying derivation to both sides of Equation (16), we obtain (see Appendix C)

(18) $$E\left[T_{O\to B}\left(t\right)\right]=5\cdot 6^{t-1}.$$

### 3.4. Random Walk from O to E

For comparison to the random walk from O to B, we now discuss the random walk starting at node O and ending at node E (see Figure 2). We denote by $T_{O\to E}\left(t\right)$ the FPT from O to E in generation *t*. The generating function is denoted as $\Phi_{T}^{O\to E}\left(t,z\right)$. With the same method as for the random walk from O to B, we obtain the following recursion:

(19) $$\Phi_{T}^{O\to E}\left(t,z\right)=\Phi_{T}^{O\to E}\left(2,\Phi_{T}^{A\to B}\left(t-2,z\right)\right).$$

We use the initial condition

(20) $$\Phi_{T}^{O\to E}\left(2,z\right)=\frac{z(3-2z^{2})}{6-5z^{2}}.$$

By applying derivation to both sides of Equation (19), we also have

(21) $$E\left[T_{O\to E}\left(t\right)\right]=7\cdot 6^{t-2}.$$

### 3.5. Random Walk to B with the Starting Node Selected Randomly

In this section, we discuss a new problem regarding a random walk from any point to a given end point. The starting point is selected based on the stationary distribution. Here, we first introduce the stationary distribution.

In the context of a random walk, the stationary distribution (also known as the invariant distribution or equilibrium distribution) refers to a probability distribution over the states of the system that remains unchanged as time progresses, assuming that the process reaches equilibrium.

For a random walk in a finite state space (such as a Markov chain), the stationary distribution Π satisfies the following condition:

(22) Π=ΠP,

Here, *P* is the transition matrix of the Markov chain, where each entry $P_{ij}$ represents the probability of transitioning from *i* to *j*.

In the random walk on a network, we have the conclusion that

(23) $$\Pi =\left(\frac{d_{1}}{\sum_{k=1}^{N}d_{k}},\frac{d_{2}}{\sum_{k=1}^{N}d_{k}},\cdots ,\frac{d_{N}}{\sum_{k=1}^{N}d_{k}}\right).$$

where $d_{k}$ stands for the degree of the vertex *k*.

Then, we discuss the random walk to B with the starting site randomly selected according to the stationary distribution Π in Equation (23). We denote the first passage time (FPT) of this random walk in the T-fractal as $T_{\Pi \to B}\left(t\right)$ in generation *t*. Moreover, $\Phi_{T}^{\Pi \to B}\left(t,z\right)$ denotes the generating function of $T_{\Pi \to B}\left(t\right)$. We also introduce a new concept in random walks, namely the return time. This is the time at which the walker returns to the initial node. We denote the return time to B as $T_{B\to B}\left(t\right)$ in generation *t* of the T-fractal. The generating function of $T_{B\to B}\left(t\right)$ is $\Phi_{T}^{B\to B}\left(t,z\right)$. It has been found that there is a relationship between $\Phi_{T}^{\Pi \to B}\left(t,z\right)$ and $\Phi_{T}^{B\to B}\left(t,z\right)$:

(24) $$\Phi_{T}^{\Pi \to B}\left(t,z\right)=\frac{z}{1-z}\times \frac{d_{B}}{2E}\times \frac{1}{\Phi_{T}^{B\to B}\left(t,z\right)}.$$

Here, $d_{B}$ stands for the degree of node B, and *E* is the total number of edges in the network. Replacing $d_{B}=1$ and $E\left(t\right)=3^{t}$ in Equation (24), we obtain

(25) $$\Phi_{T}^{\Pi \to B}\left(t,z\right)=\frac{z}{1-z}\times \frac{1}{2\times 3^{t}}\times \frac{1}{\Phi_{T}^{B\to B}\left(t,z\right)}.$$

Regarding $\Phi_{T}^{B\to B}\left(t,z\right)$, we have the following equations (see Appendix D):

(26) $$\Phi_{T}^{B\to B}\left(t,z\right)=\frac{\Phi_{T}^{B\to B}\left(t-1,z\right)}{\Psi (\Phi_{T}^{A\to B}\left(t,z\right))}.$$

and

(27) $$\Psi \left(z\right)=\frac{\Phi_{T}^{B\to B}\left(0,z\right)}{\Phi_{T}^{B\to B}\left(1,z\right)}.$$

With the initial condition $\Phi_{T}^{B\to B}\left(0,z\right)=\frac{1}{1-z^{2}}$ and $\Phi_{T}^{B\to B}\left(1,z\right)=\frac{3-2z^{2}}{3(1-z^{2})}$ (see Appendix B), we have

(28) $$\Psi \left(z\right)=\frac{3}{3-2z^{2}}.$$

Combining this with Equations (25), (26) and (28), we have

(29) $$\Phi_{T}^{\Pi \to B}\left(t,z\right)=2\Psi \left(\Phi_{T}^{A\to B}\left(t-1,z\right)\right)\times \Phi_{T}^{\Pi \to B}\left(t-1,z\right)$$

with the initial condition

(30) $$\Phi_{T}^{\Pi \to B}\left(0,z\right)=\frac{1}{2}z\left(z+1\right).$$

By calculating the derivatives for both sides of Equation (30) and using z=1, we obtain the MFPT of this random walk:

(31) $$E\left[T_{\Pi \to B}\left(t\right)\right]=\frac{4}{5}\cdot 6^{t}+\frac{7}{10}∼\frac{4}{5}\cdot 6^{t}.$$

## 4. Random Walk on Network with Stochastic Resetting

In this section, we provide a general method for the calculation of the MFPT in a general network with stochastic resetting. Then, we study the MFPT in a general discrete-time random walk with a fixed resetting rate in a general network. The findings presented in this section will aid in the determination of a random walk with stochastic resetting in a specific network, such as the T-fractal, which we discuss in this work.

### 4.1. MFPT for the Discrete-Time First Passage Process Under Resetting

Random walks with stochastic resetting are characterized by restarts occurring at a random time. We suppose that the resetting occurs at a random time *R*. Let $T_{R}$ refer to the first passage time under resetting and *T* refer to the first passage time without resetting. We obtain [39,40]

(32) $$T_{R}=\left\{\begin{array}{cc} T & T<R\\ R+T_{R}^{\prime } & T\geq R \end{array}\right.,$$

Then, in this section, we focus on the discrete-time random walk on a network with resetting. We investigate the relationship between the mean of $T_{R}$ and the generating function of *T* and *R*. Then, we calculate the mean of $T_{R}$ by using the generating function. This method was presented in Ref. [40]. Let

(33) $$I\left(T\geq R\right)=\left\{\begin{array}{cc} 0 & T<R\\ 1 & T\geq R \end{array}\right.,$$

so that we can rewrite Equation (19) as

(34) $$T_{R}=\min\left(T,R\right)+I\left(T\geq R\right)\times T_{R}^{\prime }.$$

Let E[ξ] denote the first moment (i.e., the mathematical expectation) of the random variable ξ and Pr(A) denote the probability that the event *A* will occur. Consequently, we obtain

(35) $$\begin{array}{cc} E[T_{R}] & =E\left[\min\left(T,R\right)\right]+E\left[I\left(T\geq R\right)\cdot T_{R}^{\prime }\right]\\ \; & =E\left[\min\left(T,R\right)\right]+\mathrm{Pr}\left(T\geq R\right)\cdot E\left[T_{R}^{\prime }\right]\\ \; & =E\left[\min\left(T,R\right)\right]+\left[1-\mathrm{Pr}\left(T<R\right)\right]\cdot E\left[T_{R}\right]. \end{array}$$

Note that the random variables $T_{R}$ and $T_{R}^{\prime }$ are independent and identically distributed. Of course, their first moments are equivalent. Thus, we can obtain

(36) $$E\left[T_{R}\right]=\frac{E[\min(T,R)]}{\mathrm{Pr}(T<R)}.$$

Through the definition of the first moment and some properties, we can obtain the following:

(37) $$\begin{array}{cc} E[\min(T,R)] & =\sum_{m=0}^{\infty }\mathrm{Pr}\left(\min\left(T,R\right)>m\right)\\ \; & =\sum_{m=0}^{\infty }\left(\sum_{k=m+1}^{\infty }\mathrm{Pr}\left(T=k\right)\sum_{l=m+1}^{\infty }\mathrm{Pr}\left(R=l\right)\right), \end{array}$$

(38) $$\mathrm{Pr}\left(T<R\right)=\sum_{m=0}^{\infty }\left[\mathrm{Pr}\left(T=m\right)\sum_{l=m+1}^{\infty }\mathrm{Pr}\left(R=l\right)\right].$$

### 4.2. MFPT for a Random Walk on a Network with a Fixed Resetting Rate

Now, we discuss the discrete random walk on a network with stochastic resetting at a fixed rate. At each step, there is a fixed probability γ of resetting to the initial site, and we use γ to denote the resetting rate. Meanwhile, there is a probability 1−γ of the walker walking to the nodes that are adjacent to the node that they currently occupy. Thus, the random variable *R* discussed above, which is the time taken by the walker to restart at the initial site, follows a geometric distribution with the parameter γ. In other words, for any l≥1,

(39) $$\mathrm{Pr}\left(R=l\right)={\left(1-\gamma \right)}^{l-1}\gamma .$$

This can be explained as follows: if the walker resets at time *l*, it means that they did not reset in the previous (l−1) steps (with a certain probability), and they reset at time *l* with another probability γ. Therefore, for any m≥0,

(40) $$\sum_{l=m+1}^{\infty }\mathrm{Pr}\left(R=l\right)=\sum_{l=m+1}^{\infty }{\left(1-\gamma \right)}^{l-1}\gamma ={\left(1-\gamma \right)}^{m}.$$

Substituting Equation (27) into Equations (24) and (25), we have

(41) $$\begin{array}{ccc} E[\min(T,R)] & = & \sum_{m=0}^{\infty }\left[{\left(1-\gamma \right)}^{m}\sum_{k=m+1}^{\infty }\mathrm{Pr}\left(T=k\right)\right]\\ & = & \sum_{k=1}^{\infty }\left[\mathrm{Pr}\left(T=k\right)\sum_{m=0}^{k-1}{\left(1-\gamma \right)}^{m}\right]\\ & = & \sum_{k=1}^{\infty }\left[\mathrm{Pr}\left(T=k\right)\frac{1-{\left(1-\gamma \right)}^{k}}{\gamma }\right]\\ & = & \frac{1}{\gamma }\left[1-\sum_{k=0}^{\infty }\left[\mathrm{Pr}\left(T=k\right){\left(1-\gamma \right)}^{k}\right]\right]\\ & = & \frac{1-\Phi_{T}\left(1-\gamma \right)}{\gamma }. \end{array}$$

Moreover, we have

(42) $$\begin{array}{ccc} \mathrm{Pr}(T<R) & = & \sum_{m=0}^{\infty }\left[\mathrm{Pr}\left(T=m\right){\left(1-\gamma \right)}^{m}\right]\\ & = & \Phi_{T}\left(1-\gamma \right). \end{array}$$

where $\Phi_{T}\left(z\right)$ is the generating function of the random variable T, which can be written as

(43) $$\begin{array}{c} \Phi_{T}\left(z\right)=\sum_{k=0}^{\infty }\left[\mathrm{Pr}\left(T=k\right)z^{k}\right]. \end{array}$$

Inserting Equations (28) and (29) into Equation (23), we reach an important conclusion:

(44) $$\begin{array}{c} E\left[T_{R}\right]=\frac{1-\Phi_{T}\left(1-\gamma \right)}{\gamma \Phi_{T}\left(1-\gamma \right)}. \end{array}$$

This formula can be used to calculate the mean first passage time (MFPT) with stochastic resetting. Through this formula, we can transform the problem of solving the MFPT with resetting into the problem of solving the generating function without resetting.

In Section 2, we found some recursions regarding the generating function of the random walk on a T-fractal without resetting. Meanwhile, in this section, we have found a relationship between the MFPT of the random walk with stochastic resetting at a fixed resetting rate and the generating function without resetting. Thus, in the next section, we combine these two sets of findings and obtain the MFPT of the random walk on a T-fractal with stochastic resetting.

## 5. Random Walk on T-Fractal with Stochastic Resetting

In this section, we discuss the MFPT for different random walks on the T-fractal. We consider different resetting sites, and the resetting site is the starting site. For each random walk, we discuss the optimal γ to ensure the minimum MFPT. The results presented in this section provide a resetting strategy to enhance the search efficiency, as measured via the MFPT. Let us start with a random walk from O to B with resetting.

### 5.1. Random Walk with Resetting from O to B

We now discuss the random walk on a T-fractal with resetting from O to B. The walker starts the random walk from node O, and the end node is B. At each step, there is a probability γ of the worker resetting to the initial site O, and there is a probability 1−γ of the walker walking through the adjacent node. By solving Equation (16) with the initial condition (17), we can obtain the generating function for the first passage time from nodes O to B without resetting. The result is

(45) $$\begin{array}{ccc} \Phi_{T}^{O\to B}\left(t,z\right) & = & \frac{(2^{2^{t-1}+t-4})z}{\left(3^{2^{t}-1}\times 2^{2^{t-1}+t-3}+2^{2^{t}+2t-7}\right)z^{2}+3^{2^{t}-1}}. \end{array}$$

Let $E(T_{O\to B}^{R}\left(t\right))$ denote the first moment of the random walk from O to B with resetting in generation t. Thus, using Equation (31), we have

(46) $$\begin{array}{cc} E[T_{O\to B}^{R}\left(t\right)] & =\frac{1-\Phi_{T}^{O\to B}\left(t,1-\gamma \right)}{\gamma \phi_{T}^{O\to B}\left(t,1-\gamma \right)}\\ \; & =\frac{\left(3^{2^{t}-1}\times 2^{2^{t-1}+t-3}+2^{2^{t}+2t-7}\right)\gamma^{2}-\left[2\left(3^{2^{t}-1}\times 2^{2^{t-1}+t-3}+2^{2^{t}+2t-7}\right)+2^{2^{t-1}+t-4}\right]\gamma }{2^{2^{t-1}+t-4}-2^{2^{t-1}+t-4}\gamma }\\ \; & \;+\frac{3^{2^{t-1}}-2^{2^{t-1}+t-4}}{2^{2^{t-1}+t-4}-2^{2^{t-1}+t-4}\gamma }. \end{array}$$

By taking the first-order derivative with respect to γ on both sides of Equation (46), and by setting $\frac{\partial }{\partial \gamma }E\left[T_{O\to B}^{R}\left(t\right)\right]=0$, we have

(47) $$\begin{array}{ccc} & & \left[\left(3^{2^{t-1}}\times 2^{2^{t-1}+t-3}+2^{2^{t}+2t-7}\right)2^{2^{t-1}+t-4}+2^{2^{t}+2t-8}\right]\gamma^{2}\\ & & +[2^{2^{t}+2t-7}-2\left(3^{2^{t-1}}\times 2^{2^{t-1}+t-3}+2^{2^{t}+2t-7}+3^{2^{t}-1}\right)2^{2^{t-1}-t-4}]\gamma \\ & & +[\left(3^{2^{t-1}}\times 2^{2^{t-1}-t-3}+2^{2^{t}-2t-7}+3^{2^{t}-1}\right)2^{2^{t-1}-t-4}-2^{2^{t}+2t-8}]=0. \end{array}$$

Thus, we can obtain the root (the negative root is removed).

**Figure 4:**
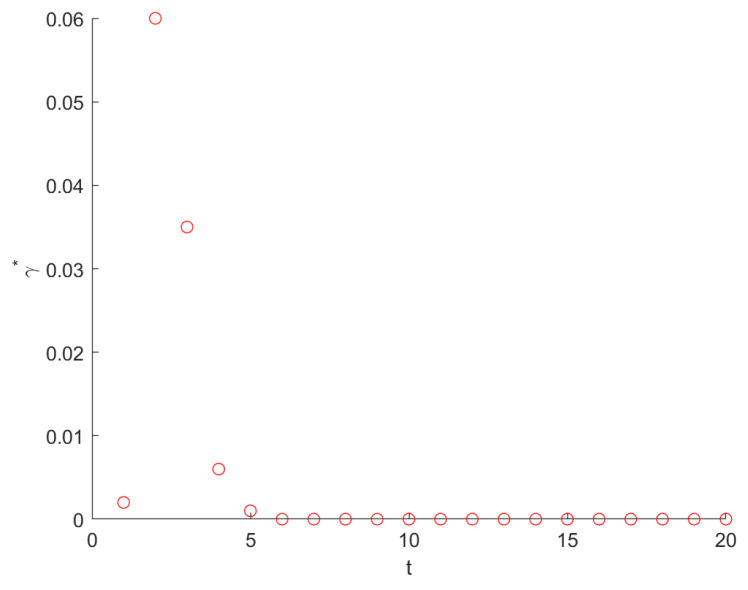
Plot of the optimal resetting probability $\gamma_{O\to B}^{*}$ as a function of t. From t = 2, the $\gamma^{*}$ decreases, and, when t →∞, $\gamma^{*}\to 0$.

By plotting $\gamma_{O\to B}^{*}$ as a function of t (see Figure 4), we find, for any generation t, the optimal solution of Equation (47) $\gamma^{*}\in \left(0,1\right)$. We also find that, from t=2, $\gamma^{*}$ decreases. Moreover, when t→∞, $\gamma^{*}\to 0$.

**Figure 5:**
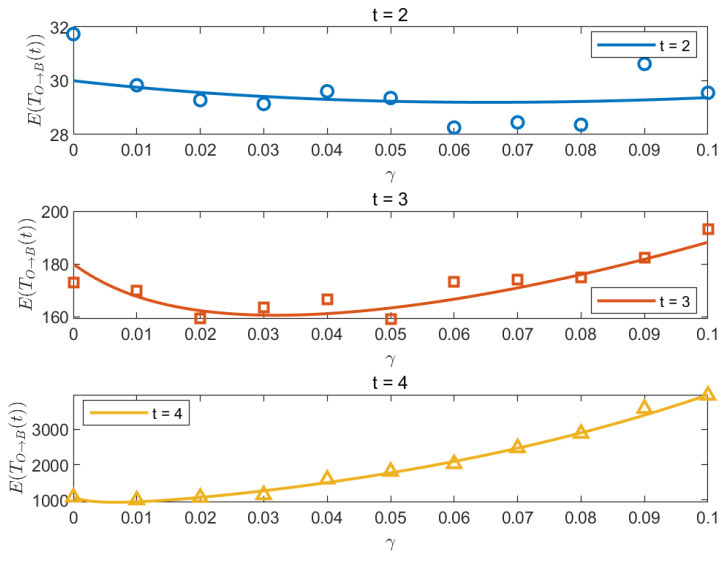
Plot of $E(T_{R})$ vs. γ when t=2,3,4; the optimal $\gamma^{*}$ is around 0.06, 0.035, and 0.008, respectively. The Monte Carlo numerical simulations are presented in symbols.

Additionally, Figure 5 shows the MFPT of the random walk from *O* to *B* with resetting rate γ for t=2,3,4. The horizontal solid lines and symbols represent the theoretical and simulation results, respectively. The Monte Carlo numerical simulations are in relatively good agreement with the theoretical solution. This shows that when the network size is very large, resetting cannot improve the efficiency of the random search from the central site O to the outermost side.

### 5.2. Random Walk with Resetting from O to E

We now discuss the random walk with resetting from O to E. At each step, there is a probability γ of the walker resetting to the initial position O. Moreover, there is a probability 1−γ of the walker walking to the adjacent node from the current position. By solving Equation (19) with the initial condition (20), we obtain the generating function of $T_{O\to E}\left(t\right)$ without resetting:

(48) $$\Phi_{T}^{O\to E}\left(t,z\right)=\frac{\left(9\times 2^{t-4}-2^{t-1}\times 3^{2t^{2}-5t+6}\right)z^{2}}{4^{t-1}-\left(2^{2t-3}\times 3^{2t^{2}-6t+8}\right)z+4^{t}\times 27^{{\left(t-2\right)}^{2}}z^{2}}.$$

Thus, the MFPT with resetting can be calculated as

(49) $$\begin{array}{cc} E[T_{O\to E}^{R}\left(t\right)] & =\frac{1-\Phi_{T}^{O\to E}\left(t,1-\gamma \right)}{\gamma \Phi_{T}^{O\to E}\left(t,1-\gamma \right)}\\ \; & =\frac{4^{t-1}-\left(2^{2t-3}\times 3^{2t^{2}-6t+8}\right)\left(1-\gamma \right)+4^{t}\times 27^{{\left(t-2\right)}^{2}}{\left(1-\gamma \right)}^{2}}{\gamma \left(9\times 2^{t-4}-2^{t-1}\times 3^{2t^{2}-5t+6}\right){\left(1-\gamma \right)}^{2}}\\ \; & \;-\frac{\left(9\times 2^{t-4}-2^{t-1}\times 3^{2t^{2}-5t+6}\right){\left(1-\gamma \right)}^{2}}{\gamma \left(9\times 2^{t-4}-2^{t-1}\times 3^{2t^{2}-5t+6}\right){\left(1-\gamma \right)}^{2}}. \end{array}$$

By taking the derivative in Equation (49) and letting $\frac{d}{d\gamma }E\left[T_{O\to E}^{R}\left(t\right)\right]=0$, we have

(50) $$\begin{array}{ccc} & & \left[4^{t}\times 27^{{\left(t-2\right)}^{2}}-\left(9\times 2^{t-4}-2^{t-1}\times 3^{2t^{2-5t+6}}\right)\right]\gamma^{2}\\ & & +[2^{2t-3}\times 3^{t^{2-6t+8}}-2\times 4^{t}\times 27^{{\left(t-2\right)}^{2}}\\ & & +(9\times 2^{t-3}-2^{t-1}\times 3^{2t^{2-5t+6}})]\gamma \\ & & +4^{t-1}+9\times 2^{t-4}-2^{t-1}\times 3^{2t^{2-5t+6}}=0. \end{array}$$

**Figure 6:**
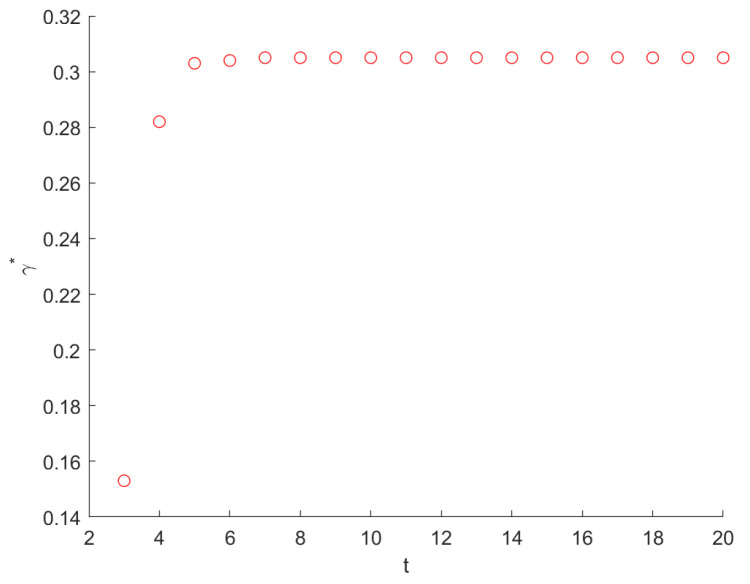
Plot of the optimal resetting probability $\gamma_{O\to E}^{*}$ as a function of *t*. When t→∞, $\gamma_{O\to E}^{*}\to 0.305$.

**Figure 7:**
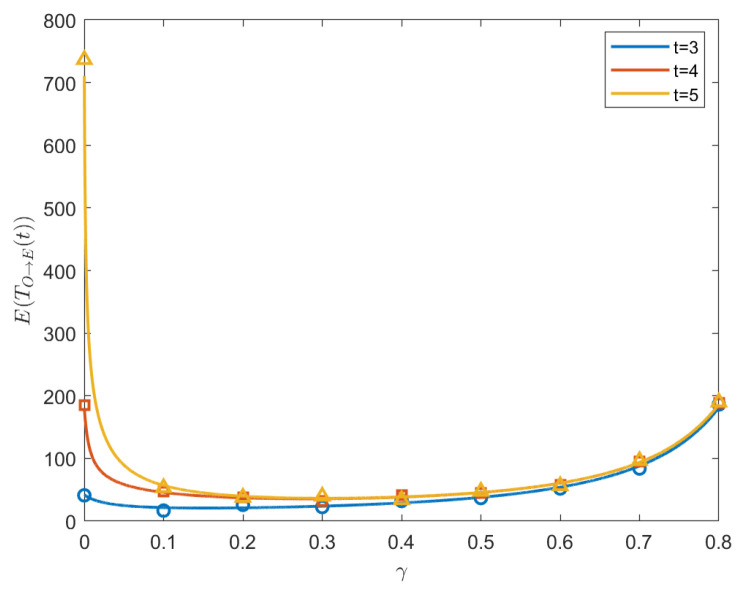
Plot of the MFPT of generations 3, 4, and 5 as a function of γ from O to E; the optimal $\gamma^{*}$ is approximately 0.15, 0.28, and 0.3, at which the MFPT reaches its minimum. The horizontal solid lines and symbols represent the theoretical and simulation results, respectively.

By solving this equation, we can derive the optimal $\gamma^{*}$. For the plot of $\gamma^{*}$ vs. *t*, see Figure 6. Additionally, Figure 7 shows the MFPT vs. γ within the interval [0,1) when t=3,4,5. We also perform a Monte Carlo simulation to obtain theoretical results; see the symbols in Figure 7.

Thus, in this situation, resetting can also improve the efficiency of the random search.

### 5.3. MFPT for a Random Walk on a T-Fractal with the Resetting Position Selected Randomly

In this section, we discuss the random walk from a random starting node to the fixed node B. At each step, there is a probability γ that the walker resets to the initial position. The starting node is selected according to the stationary distribution, as mentioned in Section 3. By solving Equation (29) with the initial condition (30), we obtain

(51) $$\begin{array}{ccc} \Phi_{T}^{\Pi \to B}\left(t,z\right) & = & \frac{2^{t-1}\times 3^{3t-2}z^{2}+2^{t-1}\times 3^{3t-2}z}{3^{2^{t-1}+t}-2\left(t+1\right)3^{t}z^{2}}. \end{array}$$

Therefore, the MFPT under resetting is

(52) $$\begin{array}{ccc} E[T_{\Pi \to B}^{R}\left(t\right)] & = & \frac{1-\Phi_{T}^{\Pi \to B}\left(t,1-\gamma \right)}{\gamma \Phi_{T}^{\Pi \to B}\left(t,1-\gamma \right)}\\ & = & \frac{\left[2\left(t+1\right)3^{t}-2^{t-1}3^{3t-2}\right]\gamma^{2}+\left[4\left(t+1\right)3^{t}+2^{t-1}3^{3t-1}\right]\gamma +3^{2^{t-1}+t}-2\left(t+1\right)3^{t}}{2^{t-1}\times 3^{3t-2}\left(\gamma^{3}-3\gamma^{2}+2\gamma +1\right)}. \end{array}$$

By taking the first-order derivative for both sides of Equation (52) and setting $\frac{d}{d\gamma }E\left[T_{\Pi \to B}^{R}\left(t\right)\right]=0$, we obtain

(53) $$\begin{array}{ccc} & & \left[4\left(t+1\right)3^{t}-2^{t-1}\times 3^{3t-2}\right]\times 2^{t-1}\times 3^{3t-2}\gamma^{2}\\ & & +\left[3^{2t-1+t}-2\left(t+1\right)3^{t}\right]\times 2^{t}\times 3^{3t-2}\gamma \\ & & +\left[2\left(t+1\right)3^{t}-3^{2t-1+t}\right]\times 2^{t-1}\times 3^{3t-2}=0. \end{array}$$

**Figure 8:**
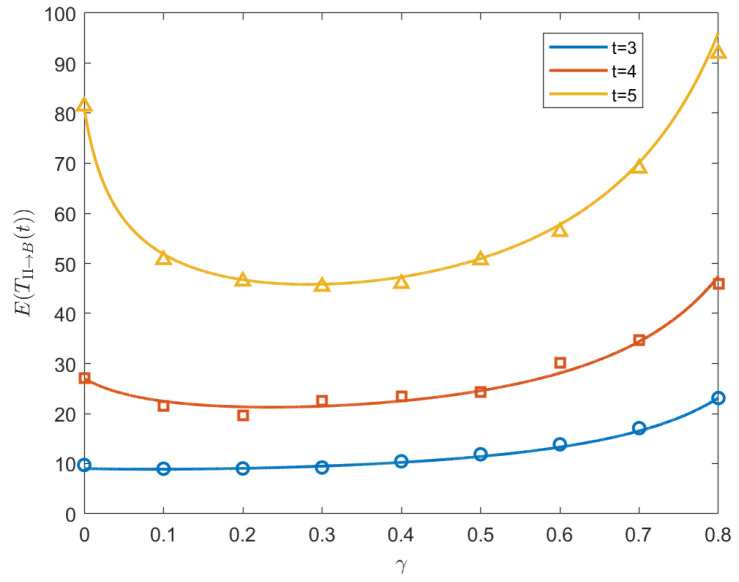
Plot of the MFPT of generations 3, 4, 5 as a function of γ from Π to B; the optimal $\gamma^{*}$ is approximately 0.08, 0.24, and 0.28, at which the MFPT reaches its minimum. The Monte Carlo simulations are presented by the symbols.

**Figure 9:**
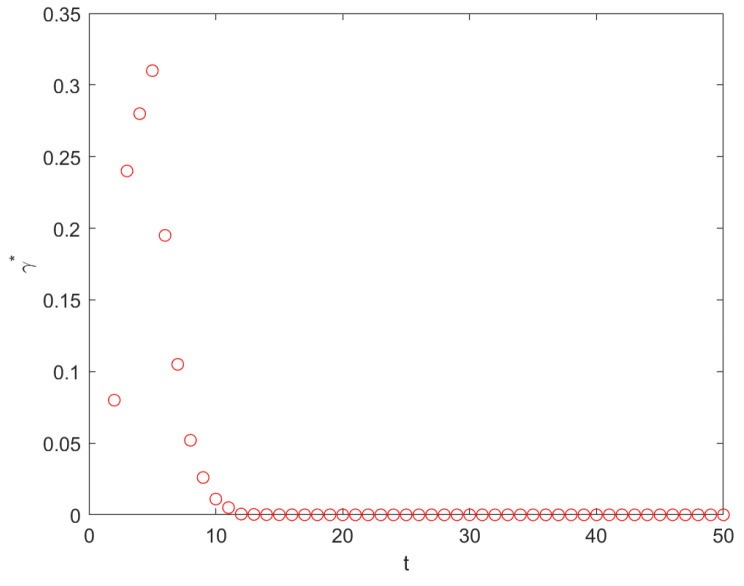
Plot of the optimal resetting probability $\gamma_{\Pi \to B}^{*}$ as a function of *t*. The $\gamma_{\Pi \to B}^{*}$ decreases from generation 3 and when t→∞, $\gamma_{\Pi \to B}^{*}\to 0$.

By solving Equation (53), we obtain the expression for the optimal $\gamma_{\Pi \to B}^{*}$. Figure 8 shows the MFPT vs. γ within the interval [0,1) when t=3,4,5. We also perform the Monte Carlo simulation to validate the theoretical results; see the symbols in Figure 8. Additionally, Figure 9 plots the optimal $\gamma_{\Pi \to B}^{*}$ as a function of generation *t*. We find that, from generation 4, the optimal $\gamma_{\Pi \to B}^{*}$ decreases monotonically with the increase in the generation *t*. Moreover, when t→∞, $\gamma_{\Pi \to B}^{*}\to 0$.

Thus, we conclude that while applying resetting to a randomly drawn value from the stationary distribution can improve efficiency in smaller networks, as network size increases significantly, the optimal resetting rate approaches zero, indicating that resetting does not enhance the efficiency of random searches in very large networks. The conclusions obtained in this section suggest limitations to the effectiveness of resetting strategies for T-fractals and other extensive networks.

## 6. Conclusions

In this study, we explored the dynamics of a random walk on a T-fractal with stochastic resetting, focusing on the effects of resetting on the search efficiency of the random walker. By employing the generating function technique, we established a recursive relation between the generating functions of the first passage time (FPT) and derived the connection between the mean first passage time (MFPT) with resetting and the generating function of the FPT without resetting. This analytical framework allowed us to gain important insights into the ways in which stochastic resetting influences the MFPT.

We examined various scenarios in which a random walker reached a target site from the starting position, identifying the optimal resetting probability $\gamma^{*}$ for each case. This optimal $\gamma^{*}$ minimizes the MFPT, thereby enhancing the efficiency of the search process. Our findings indicate that, in certain scenarios, compared with the MFPT without resetting, the introduction of a resetting mechanism can significantly improve search efficiency, particularly in cases where the network model size is moderate.

This work demonstrates the potential of stochastic resetting as a valuable strategy for the optimization of search processes in complex networks. By minimizing the time taken to locate a target, resetting can be particularly beneficial in applications where quick and efficient searches are crucial. Our results contribute to the broader understanding of stochastic processes on fractal structures and provide a foundation for further exploration into the optimization of search strategies in random environments.

## Data Availability

Data is contained within the article.

## Abbreviations

The following abbreviations are used in this manuscript:

MFPT	Mean first passage time
FFP	First passage probability
FPT	First passage time

## Appendix A. Some Basic Properties of the Generating Function

Let ξ be a discrete random variable that takes only non-negative integer values. Suppose that the probability distribution of the random variable ξ is $p_{n}$, which means that

(A1) $$\mathrm{Pr}\left(\xi =n\right)=p_{n}\;n=0,1,2,\ldots$$

The probability generating function of ξ is defined as

(A2) $$\Phi_{\xi }\left(z\right)=\sum_{n=0}^{\infty }p_{n}z^{n}.$$

The definition of the probability generating function $\Phi_{\xi }\left(z\right)=\sum_{n=0}^{\infty }p_{n}z^{n}$ is grounded in the foundational principles of generating functions in probability theory. This definition is not arbitrary; rather, it leverages the ability of generating functions to encode the probability distribution $\left\{p_{n}\right\}$ of a discrete random variable ξ into a single analytic function.

As shown in the above formula, the generating function is determined by the probability distribution. Therefore, we can infer the probability distribution based on the Taylor expansion of the generating function at z=0:

(A3) $$p_{n}=\frac{{\left(\partial^{n}\Phi_{\xi }\left(z\right)/\partial z^{n}\right)}_{z=0}}{n!}.$$

Moreover, we can derive the mathematical expectation of the random variable ξ through derivatives of $\Phi_{\xi }\left(z\right)$ calculated at z=1:

(A4) $$E\left[\xi \right]={\left(\frac{\partial \Phi_{\xi }\left(z\right)}{\partial z}\right)}_{z=1}.$$

Finally, we list two important properties of the generating function, which are helpful for the calculation of the generating function of the random walk.

If $\xi_{1}$ and $\xi_{2}$ are two random variables whose generating functions are $\Phi_{\xi_{1}}\left(z\right)$ and $\Phi_{\xi_{2}}\left(z\right)$, respectively, then the generating function of $\xi_{1}+\xi_{2}$ is

(A5) $$\Phi_{\xi_{1}+\xi_{2}}\left(z\right)=\Phi_{\xi_{1}}\left(z\right)\Phi_{\xi_{2}}\left(z\right).$$

Let *N*, $\xi_{1}$, $\xi_{2}$, … be independent random variables and $\xi_{i}\left(i=1,2,\ldots ,N\right)$ be identically distributed; the generating functions of $\xi_{i}$ are all $\Phi_{\xi }\left(z\right)$, and the generating function of *N* is $\Phi_{N}\left(z\right)$. The random variable $S_{N}$ defined as

(A6) $$S_{N}=\sum_{i=1}^{N}\xi_{i}.$$

Then, the generating function of $S_{N}$ is

(A7) $$\Phi_{S_{N}}\left(z\right)=\Phi_{N}\left(\Phi_{\xi }\left(z\right)\right).$$

## Appendix B. The Calculation of the Initial Condition

For a small network, the probability generating function can be calculated directly by using the symbolic toolbox of MATLAB. Below are the general methods used to calculate the initial condition.

First, let $M=(P_{ij})$ be the transition matrix of the random walk on the network, where

(A8) $$P_{ij}=\left\{\begin{array}{cc} \frac{1}{d_{i}} & \mathrm{if}\;i\;\mathrm{links}\;\mathrm{with}\;j\;\mathrm{and}\;i\;\mathrm{is}\;\mathrm{not}\;\mathrm{in}\;\mathrm{the}\;\mathrm{absorbing}\;\mathrm{domain}\\ 0 & \mathrm{otherwise} \end{array}\right..$$

Then, the probability generating function of the matrix M can be obtained:

(A9) $$\Pi \left(z\right)=\sum_{n=0}^{\infty }{\left(zM\right)}^{n}={\left(I-zM\right)}^{-1}$$

where I is the identity matrix, and $\Pi \left(z\right)=(\Phi_{ij}\left(z\right))$, where $\Phi_{ij}\left(z\right)$ represents the random walk starting at node *i* and ends at node *j*. Moreover, $\Phi_{ii}\left(z\right)$ refers to the return time to node *i*. If *j* is in the absorbing domain, then $\Phi_{ij}\left(z\right)$ can be referred to as the generating function of the first passage time from *i* to *j*. Meanwhile, $\Phi_{ii}\left(z\right)$ refers to the first return time to node *i*.

In the context of the T-fractal network, in generation 1, we now consider the generating function of the first passage time from nodes A to B, denoted as $\Phi_{T}^{A\to B}\left(1,z\right)$. First, the transition matrix can be written as follows (where node B is the absorbing node):

(A10) $$M_{1}=\left(\begin{array}{cccc} 0 & 0 & 0 & 1\\ 0 & 0 & 0 & 0\\ 0 & 0 & 0 & 1\\ \frac{1}{3} & \frac{1}{3} & \frac{1}{3} & 0 \end{array}\right).$$

Here, the nodes A, B, C, O are denoted by the numbers 1, 2, 3, 4. Then, we use MATLAB to calculate Π(z), and the generating function of the random walk from A to B is

(A11) $$\Phi_{T}^{A\to B}\left(1,z\right)=\Pi \left(1,2\right)=\frac{z^{2}}{3-2z^{2}}.$$

Moreover, we can calculate the generating function from O to B:

(A12) $$\Phi_{T}^{O\to B}\left(1,z\right)=\Pi \left(4,2\right)=\frac{z}{3-2z^{2}}.$$

In order to calculate the return time to node B without a trap, we can rewrite the matrix for the situation without a trap:

(A13) $$M_{2}=\left(\begin{array}{cccc} 0 & 0 & 0 & 1\\ 0 & 0 & 0 & 1\\ 0 & 0 & 0 & 1\\ \frac{1}{3} & \frac{1}{3} & \frac{1}{3} & 0 \end{array}\right).$$

Moreover, by calculating the Π(z) in Equation (A9), we obtain

(A14) $$\Phi_{T}^{B\to B}\left(1,z\right)=\Pi \left(2,2\right)=\frac{3-2z^{2}}{3(1-z^{2})}.$$

Through the definition of the generating function in Equation (A2), it is obvious that

(A15) $$\Phi_{T}^{B\to B}\left(0,z\right)=\frac{1}{1-z^{2}}.$$

For the following calculation, we define

(A16) $$\Psi \left(z\right)=\frac{\Phi_{T}^{B\to B}\left(0,z\right)}{\Phi_{T}^{B\to B}\left(1,z\right)}=\frac{3}{3-2z^{2}}.$$

For generation 2, we can also use the same method to calculate $\Phi_{T}^{D\to E}\left(2,z\right)$. First, we write the transition matrix of generation 2 where E is the absorbing domain:

(A17) $$M_{3}=\left(\begin{array}{cccccccccc} 0 & 0 & 0 & \frac{1}{3} & \frac{1}{3} & \frac{1}{3} & 0 & 0 & 0 & 0\\ 0 & 0 & 0 & 0 & 1 & 0 & 0 & 0 & 0 & 0\\ 0 & 0 & 0 & 0 & 0 & 1 & 0 & 0 & 0 & 0\\ 0 & 0 & 0 & 0 & 0 & 0 & 1 & 0 & 0 & 0\\ \frac{1}{3} & \frac{1}{3} & 0 & 0 & 0 & 0 & 0 & \frac{1}{3} & 0 & 0\\ 0 & 0 & 0 & 0 & 0 & 0 & 0 & 0 & 0 & 0\\ \frac{1}{3} & 0 & 0 & \frac{1}{3} & 0 & 0 & 0 & 0 & 0 & \frac{1}{3}\\ 0 & 0 & 0 & 0 & 1 & 0 & 0 & 0 & 0 & 0\\ 0 & 0 & 0 & 0 & 0 & 1 & 0 & 0 & 0 & 0\\ 0 & 0 & 0 & 0 & 0 & 0 & 1 & 0 & 0 & 0 \end{array}\right)$$

Then, we can derive the initial condition as

(A18) $$\Phi_{T}^{D\to E}\left(2,z\right)=\Pi \left(5,6\right)=\frac{z^{2}}{6-5z^{2}}.$$

and

(A19) $$\Phi_{T}^{O\to E}\left(2,z\right)=\Pi \left(1,6\right)=\frac{z(3-2z^{2})}{6-5z^{2}}.$$

## Appendix C. The Derivation of Equations (10), (14) and (18) in Section 3

Let us first introduce some notations to facilitate the subsequent discussion. First, we denote by $E[T_{A\to B}\left(t\right)]$ the first moment of the random variable FPT from nodes A to B in generation *t*. According to Equation (A4), it is obvious that

(A20) $$E\left[T_{A\to B}\left(1\right)\right]={\left(\frac{d}{dz}\Phi_{T}^{A\to B}\left(1,z\right)\right)}_{z=1}=6.$$

In the section on the random walk from A to B, we found the recursion

(A21) $$\Phi_{T}^{A\to B}\left(t,z\right)=\Phi_{T}^{A\to B}\left(1,\Phi_{T}^{A\to B}\left(t-1,z\right)\right).$$

Now, we take the derivative on both sides of this equation and combine it with Equation (A4), setting z=1. We obtain

(A22) $$\begin{array}{ccc} E[T_{A\to B}\left(t\right)] & = & {\left(\frac{d}{dz}\Phi_{T}^{A\to B}\left(1,z\right)\right)}_{z=1}\cdot {\left(\frac{\partial }{\partial z}\Phi_{T}^{A\to B}\left(t-1,z\right)\right)}_{z=1}\\ & = & {\left(E\left[T_{A\to B}\left(1\right)\right]\right)}^{t}=6^{t}. \end{array}$$

As for the random walk from D to E, the recursion is

(A23) $$\Phi_{T}^{D\to E}\left(t,z\right)=\Phi_{T}^{D\to E}\left(2,\Phi_{T}^{A\to B}\left(t-2,z\right)\right).$$

We can also take the derivatives on both sides using the same method, and we obtain

(A24) $$E\left(T_{D\to E}\left(t\right)\right)={\left(\frac{d}{dz}\Phi_{T}^{D\to E}\left(2,z\right)\right)}_{z=1}\cdot {\left(\frac{\partial }{\partial z}\Phi_{T}^{A\to B}\left(t-2,z\right)\right)}_{z=1}=2\cdot 6^{t-1}.$$

For the random walk from O to B, the recursion is

(A25) $$\Phi_{T}^{O\to B}\left(t,z\right)=\Phi_{T}^{O\to B}\left(1,\Phi_{T}^{A\to B}\left(t-1,z\right)\right).$$

Using the same method, we can obtain

(A26) $$E\left(T_{O\to B}\left(t\right)\right)={\left(\frac{d}{dz}\Phi_{T}^{O\to B}\left(1,z\right)\right)}_{z=1}\cdot {\left(\frac{\partial }{\partial z}\Phi_{T}^{A\to B}\left(t-1,z\right)\right)}_{z=1}=5\cdot 6^{t-1}.$$

(A27) $$E\left(T_{O\to E}\left(t\right)\right)={\left(\frac{d}{dz}\Phi_{T}^{O\to E}\left(2,z\right)\right)}_{z=1}\cdot {\left(\frac{\partial }{\partial z}\Phi_{T}^{A\to B}\left(t-2,z\right)\right)}_{z=1}=7\cdot 6^{t-2}.$$

## Appendix D. The Derivation of Equations (26) and (27)

Due to the symmetry of the network, the outermost nodes (such as A, B, C; see Figure 2) are topologically equivalent. Thus, without loss of generality, we only consider the return time for B. We denote by $T_{B\to B}\left(t\right)$ the return time of B in generation t of the T-fractal (note that this may not be the first return time). Moreover, we denote by $\Phi_{T}^{B\to B}\left(t,z\right)$ the generating function of $T_{B\to B}\left(t\right)$. Similarly to the method used in Section 3, we first consider an arbitrary path that starts from B and ends at B in generation t of the T-fractal. It can be written as

(A28) $$\pi =(v_{0}=B,v_{1},v_{2},\cdots ,v_{T_{B\to B}\left(t\right)}=B)$$

where $v_{i}$ is the node that the walker reaches at time i. Moreover, let Ω={A,B}, as the node generated in generation 0. Similarly to the approach applied in Section 3, we can obtain a simplified path whose nodes are in Ω:

(A29) $$\sigma \left(\pi \right)=\left(v_{\tau_{0}}=B,v_{\tau_{1}},\cdots ,v_{N}=B\right)$$

where $v_{\tau_{i}}\in \Omega$ and N stands for the return time for B on the T-fractal in generation 0. Thus, the generating function of N is $\Phi_{T}^{B\to B}\left(0,z\right)$. For any return path of B, it is possible that $v_{\tau_{N}}$ is not the last node in path π. In other words, after $v_{\tau_{N}}$, there may be a sub-path from B to B that does not reach node A. We can regard this as the return time of B with trap (or absorbing domain) A. We denote its length by $T_{B\to B}^{\prime \prime }\left(t\right)$ in generation t of the T-fractal. Moreover, the generating function of the random variable $T_{B\to B}^{\prime \prime }\left(t\right)$ is denoted as $\Phi_{T^{\prime \prime }}^{B\to B}\left(t,z\right)$. Let $T_{i}=\tau_{i}-\tau_{i-1}$ denote the time interval between the random walks in Ω, and we have

(A30) $$T_{B\to B}\left(t\right)=T_{1}+T_{2}+\cdots +T_{N}+T_{B\to B}^{\prime \prime }\left(t\right).$$

Here, $T_{i}\left(i=1,2,\cdots ,N\right)$ are independent identically distributed random variables, and each of them stands for the random walk from A to B (or B to A) in generation t of the T-fractal. Thus, the generating function of $T_{i}$ is $\Phi_{T}^{A\to B}\left(t,z\right)$. Moreover, using the property of the generating function (A5) (A7), the generating function of $T_{B\to B}\left(t\right)$ can be written as

(A31) $$\Phi_{T}^{B\to B}\left(t,z\right)=\Phi_{T}^{B\to B}\left(0,\Phi_{T}^{A\to B}\left(t,z\right)\right)\times \Phi_{T^{\prime \prime }}^{B\to B}\left(t,z\right).$$

For the random variable $T_{B\to B}^{\prime \prime }\left(t\right)$, we set Ω={A,B,C,O}, and $T_{i}$ stands for the time interval between the random walks in set Ω. We can use the same method to derive

(A32) $$T_{B\to B}^{\prime \prime }\left(t\right)=T_{1}+T_{2}+\cdots +T_{N}+T_{B\to B}^{\prime \prime }\left(t-1\right).$$

The generating function of $T_{i}$ is $\Phi_{T}^{A\to B}\left(t-1,z\right)$, and the generating function of the random variable *N* is $\Phi_{T^{\prime \prime }}^{B\to B}\left(1,z\right)$. Due to the independence of the random variables $T_{i}\left(i=1,2,\cdots ,N\right)$ and $T_{B\to B}^{\prime \prime }\left(t-1\right)$, we have

(A33) $$\Phi_{T}^{B\to B}\left(t,z\right)=\Phi_{T}^{B\to B}\left(1,\Phi_{T}^{A\to B}\left(t-1,z\right)\right)\times \Phi_{T^{\prime \prime }}^{B\to B}\left(t-1,z\right).$$

Replacing $\Phi_{T^{\prime \prime }}^{B\to B}\left(t,z\right)$ and $\Phi_{T}^{A\to B}\left(t,z\right)$ in Equation (A33), respectively, with Equation (A31), we obtain the recurrence for $\Phi_{T}^{B\to B}\left(t,z\right)$ and it can be simplified as

(A34) $$\Phi_{T}^{B\to B}\left(t,z\right)=\frac{\Phi_{T}^{B\to B}\left(t-1,z\right)}{\Psi (\Phi_{T}^{A\to B}\left(t,z\right))}$$

where

(A35) $$\Psi \left(z\right)=\frac{\Phi_{T}^{B\to B}\left(0,z\right)}{\Phi_{T}^{B\to B}\left(1,z\right)}$$

which is similar to Equations (26) and (27).
